# The hospital costs of complications following major abdominal surgery: a retrospective cohort study

**DOI:** 10.1186/s13104-024-06720-z

**Published:** 2024-02-27

**Authors:** Angelica Armellini, Shaun Chew, Samuel Johnston, Vijayaragavan Muralidharan, Mehrdad Nikfarjam, Laurence Weinberg

**Affiliations:** 1 Department of Anaesthesia, Austin Health, Heidelberg, Australia; 2 Department of Critical Care, The University of Melbourne, Austin Health, Heidelberg, Australia; 3 Department of Surgery, Austin Health, University of Melbourne, Heidelberg, Australia

**Keywords:** Postoperative complications, Major abdominal surgery, Hospital costs

## Abstract

**Objective:**

Postoperative complications following major abdominal surgeries is a pressing concern for hospital care and health economics. Given the paucity of available cost data for patients undergoing major abdominal surgery, we evaluated the number and the severity of postoperative complications following major abdominal surgeries and calculated the costs borne by a single centre university hospital within an Australian healthcare system.

**Results:**

The overall incidence of postoperative complications for 1790 adult patients undergoing major abdominal surgeries (i.e., colonic, liver, small bowel resections and Whipple procedures) between January 2013 and June 2018 was 75.2%. Of these complications, 56.9% were minor (Clavien–Dindo (CVD) Grades I or II) and 15.5% were major (CVD Grades III or IV). As the severity of complications increased, median adjusted total hospital costs rose significantly, with a median (interquartile range [IQR]) of AUD 29,519.70 (IQR 21,828.80–40,527.90) in CVD Grade II versus AUD 50,702.40 (IQR 35,866.00–69,296.80) in CVD Grade III (*p* <.001). Further, developing one, two or three complications resulted in significantly increased hospital costs by AUD 2618.30 (13.3% increase), AUD 3605.50 (16.2% increase) and AUD 3173.00 (12.3% increase) (*p <*.0001), respectively, with an exponential spike in costs incurred by patients who developed more than three complications (AUD 23,719.70; 81.7% increase; *p* < 0001).

**Supplementary Information:**

The online version contains supplementary material available at 10.1186/s13104-024-06720-z.

## Introduction

The financial aspect of healthcare is a vital factor in determining the sustainability of our healthcare system. An estimated 10% of global gross domestic product was spent on healthcare from 2018 to 2019. This figure mirrors Australia’s gross domestic product healthcare costs, amounting to 10% [1, 2]. Growing expectations to deliver high-quality care compounded by increasing healthcare costs have prompted policymakers to change the paradigm by performing high-quality economic assessments [3].

Major abdominal surgeries consist of hepato-pancreatobiliary, upper-gastrointestinal and colorectal surgeries. Although the incidence of postoperative death following major abdominal surgeries is low across general populations, these procedures carry a high overall postoperative morbidity of 35% [4]. These postoperative complications pose significant financial ramifications for our already financially constrained healthcare system.

There is limited data on the effects of the costs associated with postoperative complications following major abdominal surgeries. This paper aims to evaluate the relationship between postoperative complications (the number and the severity of complications) and hospital costs, borne by the Australian healthcare system. A retrospective data analysis was performed to compare different types of major abdominal surgeries and their associated costs and appreciate the relationship between postoperative complications and potential drivers of increased hospital costs. We hypothesised that postoperative complications are common, and that both an increase in the number and in the severity of postoperative complications would be associated with increased hospital costs.

## Materials and methods

This retrospective single-centre cohort study was conducted at the Austin Hospital, a university teaching hospital in Victoria, Australia. All patients were managed according to the standardised Enhanced Recovery After Surgery (ERAS) guidelines at the institution. All patients’ preoperative blood results were optimised based on the National Blood Authority’s patient blood management guidelines [5]. The study was reported following the Strengthening the reporting of cohort studies in surgery (STROCSS) 2019 Guideline [6].

The study included all adult (> 18 years of age) patients who underwent colonic, small bowel, liver resections and Whipple operations of any surgical technique (open and laparoscopic) and any urgency status (emergency and elective) between January 2013 and June 2018. The exclusion criteria were strategically formulated to allow accurate comparisons across specific homogenous patient cohorts and emphasise the costs associated with major abdominal surgeries.

The primary outcome was the total hospital costs associated with each surgical procedure (i.e., colorectal, liver, small bowel and Whipple procedure surgeries). The secondary outcomes included: (1) the relationship between the total associated hospital costs and the number of complications following major abdominal surgeries; and (2) the relationship between the severity of complications and the total associated hospital costs.

Complications were stratified according to the Clavien–‍Dindo (CVD) classification system, which grades complications based on the amount of intervention required [7]. The total hospital costs included the direct and indirect in-hospital expenditure of index admissions for major abdominal surgeries. Employing an activity-based costing approach, the costs are reflected based on volume of service provided. A relevant hospital cost-bucket was devised to help categorise components of the total cost. These categories consisted of the following perioperative areas - operating theatre, anaesthesia, intensive care unit (ICU), blood and blood products, medical, pathology, pharmacy, and radiology services, ward, allied health including dieticians, and physiotherapists, readmissions, and health in the home. Direct and indirect in-hospital costs were tallied for each patient. Costs were adjusted to the Australian dollar (AUD) and inflated based on the end-of-fiscal quarter Australian Consumer Price Index on 31 December 2019.

Data were extracted from the electronic medical records from the Austin Hospital. Data accounted for various parameters that were recorded during preoperative, intraoperative, and postoperative periods. These parameters included the patient demographics, American Society of Anaesthesiologists (ASA) classes, Charlson Comorbidity Index (CCI), preoperative blood results, anaesthetic agents administered, intraoperative and postoperative fluid balance and blood results, procedures performed intraoperatively, ICU length of stay, readmission within 30 days, presence of complications and grade of complications.

### Statistical analysis

Statistical analysis was performed using GraphPad Prism v9.2.0.332. Study patients were first classified into four main groups: colorectal, liver, small bowel, and Whipple procedure surgeries. They were then categorised based on the number of complications. Study patients were also grouped based on the severity of complications using the CVD classification system (i.e., Grades I, II, III, IV and V). Descriptive statistical data are presented as median and interquartile range (IQR) values, minimum and maximum values and the number of proportions and percentages where appropriate. Comparative statistics were calculated using the Kruskal-Wallis test, where a *p-*value < 0.05 was considered a significant difference.

## Results

A total of 1790 patients underwent major abdominal surgeries, of which 868 (48.5%) underwent colorectal surgeries, 422 (23.6%) underwent liver surgeries, 348 (19.4%) underwent small bowel surgeries and 152 (8.5%) underwent Whipple procedures. The median patient age was 64 years (IQR 53–74), and 56.5% of patients were male. The median body mass index (BMI) was 26 (IQR 23–30), and 58.2% of patients were classed as ASA 3 or greater. The median CCI was 5 (IQR 3–8).

Baseline patient characteristics and preoperative data, intraoperative variables, and postoperative variables by surgical type are presented in supplementary Tables 1, 2, and 3 respectively (see additional files 1, 2, and 3).

In total, 1346 (75.2%) patients developed at least one postoperative complication. The median number of complications was 2 (IQR 1–4). A summary of the number and severity of complications is outlined in supplementary Table 4 (see additional file 4). Of those patients with complications, 1018 (56.9%) were classified as CVD Grade I or II, 108 (6.0%) as CVD Grade III and 170 (9.5%) as CVD Grade IV. A total of 50 (2.8%) patients died during their index hospital admissions, classified as CVD Grade V.

The total hospital costs are presented in Table 1. The median total cost for the entire group was AUD 25,856.20 (IQR 19,000.70–39,818.50). The median total cost associated with colorectal and liver surgeries was AUD 25,019.70 (IQR 18,850.30–36,513.90) and AUD 24,601 (IQR 19,736.30–37,078.00), respectively. The median total cost from small bowel surgeries and from Whipple procedures amounted to AUD 24,124.40 (IQR 17,346.80–41,227.00), and AUD 51,571.10 (IQR 36,730.50–76,447.60), respectively.

**Table 1:** Cost of complications in Australian dollar ($) adjusted for consumer price index in patients undergoing abdominal surgery. Data is presented as median (25th:75th), Mean (SD), Min– Max values

Cost variables	Total (*n* = 1790)	Colorectal (*n* = 868)	Liver (*n* = 422)	Small Bowel (*n* = 348)	Whipple’s (*n* = 152)
Allied health cost ($)	507.8 (113:2;1159.3); 0-17963.2	526.9 (55.3:1114.8); 0-6969.8	410.6 (76.6:892.7); 0-10156.4	670 (139.5:1515.6); 0-17963.2	1511.8 (887.6:2145.1); 38.3-11938.7
Anaesthesia cost ($)	2717.4 (1864.8:3671.0); 0-27756.9	2815.9 (2046.9:3580.4); 0-14256.1	2933.7 (1841.7:3718.8); 0-17402.7	2082.1 (1601.5:2879.5); 0-17671.9	5405.1 (4051.4:6658.3); 0-27756.9
Blood products cost ($)	0 (0:64.7); 0-37011.4	0 (0:64.3); 0-14234	0 (0:30.6); 0-14721.4	0 (0:130.3); 0-37011.4	0 (0:129.8); 0-36404.2
Health in the home cost ($)	0 (0:0); 0-37662.9	0 (0:0); 0-20648	0 (0:0); 0-15900.8	0 (0:0); 0-15868.1	0 (0:0); 0-37662.9
Intensive care unit cost ($)	0 (0:2884.8); 0-183974.1	0 (0:1592.2); 0-183088.8	1857.1 (0:3106.9); 0-101155.8	0 (0:4644.2); 0-183974.1	4109.8 (2896.1:9829.6); 0-171157.4
Medical cost ($)	1878.6 (1274.6:3088.4); 130.6-78920.4	1842.7 (1313.4:2986.9); 155.5-36474.4	1483.4 (950.9:2175.4); 130.6-29514.3	2060.5 (1333.9:3426.4); 163.2-50354.7	3672.6 (2298.1:7427.4); 415.9-78920.4
MET Call cost ($)	0 (0:0); 0-2131.5	0 (0:0); 0-2131.5	0 (0:0); 0-2083.3	0 (0:0); 0-2130.1	0 (0:0); 0-916.5
Operating theatre cost ($)	8956.1 (6286.4:13055.9); 0-115504.7	8838.3 (6542:12153); 0-59304.8	10864.5 (7641.7:16616.9); 0-62055.8	6692.7 (4823.5:9644.9); 0-92332.6	14707.7 (11111.9: 29256.2); 0-115504.7
Pathology cost ($)	1020 (525.4:1474.6); 0-15566.2	1022.6 (495.7:1337); 0-10685.2	1015.7 (566.1:1586.1); 0-15566.2	834.2 (446.9:1375.8); 0-12162.9	2055.5 (1367.2:2966.3); 572.4-13922.4
Pharmacy cost ($)	377.7 (244.6:672); 0-79377.9	366.4 (248.5:584.4); 0-24233.6	369.5 (261.9:495.4); 0-51729.1	347.2 (175.9:1097.6); 0-79377.9	861.9 (550.4:1803.9); 0-12543.6
Radiology cost ($)	194 (0:955.8); 0-25333.3	98.4 (0:784); 0-14461.9	232.8 (108:877.3); 0-25333.3	257.3 (0:1089.2); 0-15857.8	931.7 (233: 1916.1); 66.5-14131.3
Readmission cost ($)	0 (0:0); 0-195896.9	0 (0:0); 0-195896.9	0 (0:0); 0-39721.4	0 (0:0); 0-110217.7	0 (0:0); 0-26139.6
Ward cost ($)	5927.6 (4374.5:9372.6); 65.2-222883.6	6101.6(4638.7:9373.1); 65.2-222883.6	4592 (3634.7:6243.3); 648.2-97150.8	6273.7 (4417.2:10805.2); 70.1-149567.9	9475.5 (6911.2:17947.1); 1030.7-78790.5
Grand total ($)	25856.2 (19000.7:39818.5); 4477.4-629137.2	25019.7 (18850.3:36513.9); 5866.0-321404.5	24601.9 (19736.3:37078.0); 4477.4-396457.17	24124.4 (17346.8:41227.0); 7083.3-629137.2	51571.1 (36730.5:76447.6); 10124.5-477157.0

**Fig. 1:**
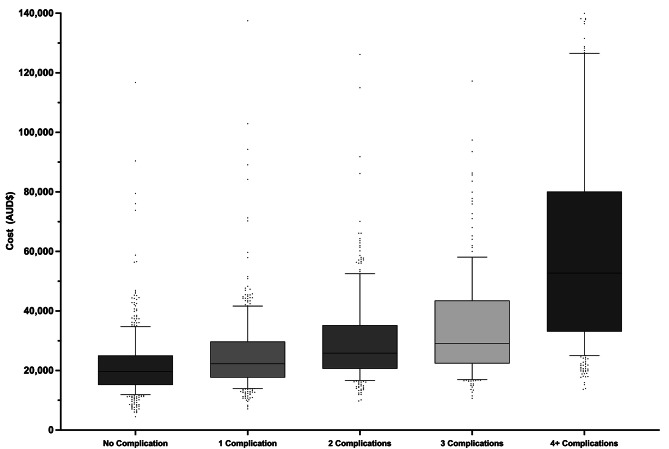
Number of complications vs. Cost (AUD$)

There was a significant difference in the total costs when comparing the five groups of patients (i.e., no complications, one complication, two complications, three complications and more than three complications) (*p* <.0001). All cost parameters increased significantly as the number of complications increased (*p* <.0001), including readmission costs (*p* =.0068). All cost variables for the number of complications are reflected in Fig. 1 and supplementary Table 5 (see additional file 5).

**Fig. 2:**
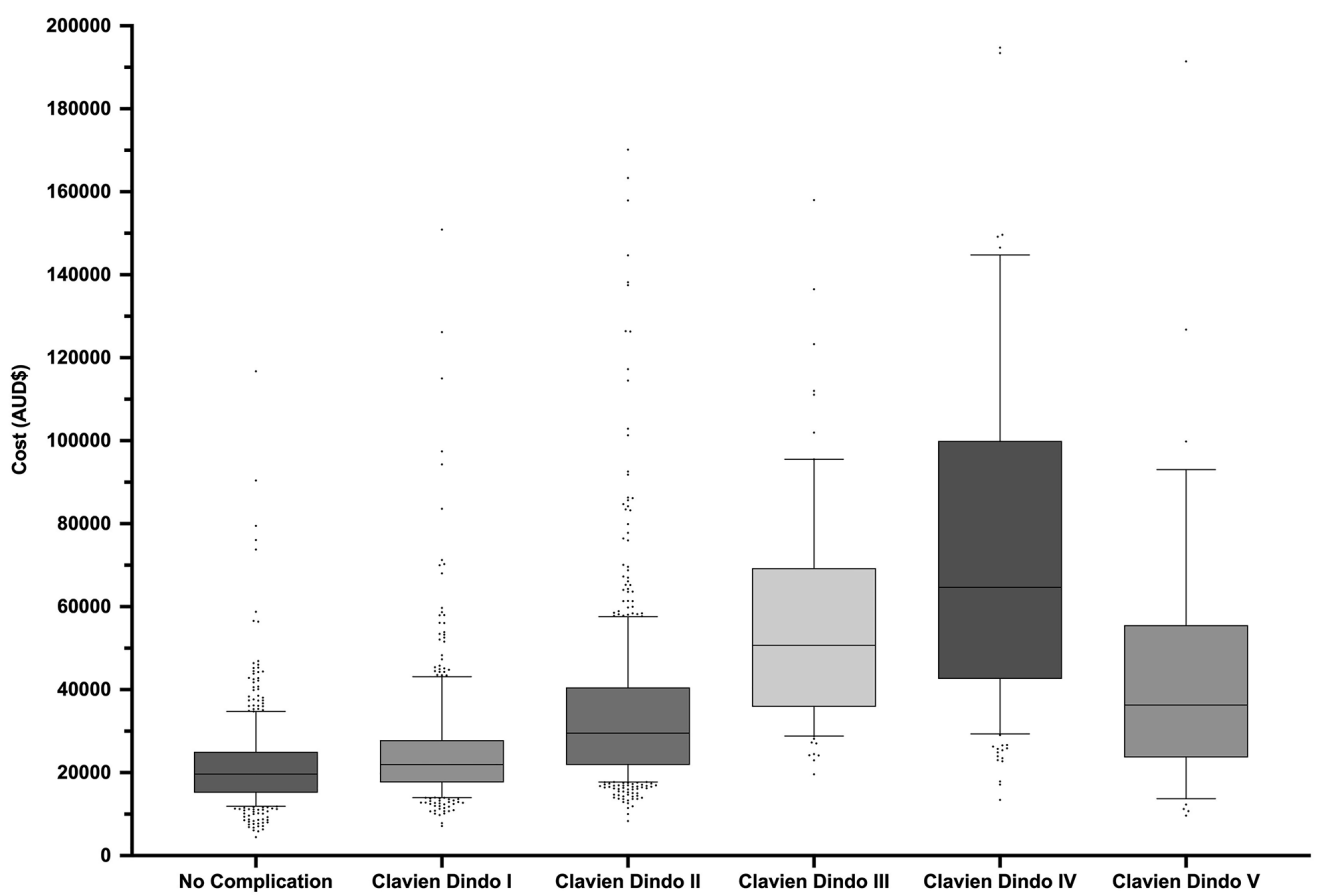
Severity of complications (Clavien-Dindo Grade) vs. Cost (AUD$)

As the CVD grading of complications increased (Grade I to IV), there was a significant increase in total hospital costs (*p* <.0001). The median total costs decreased significantly from Grade IV patients to Grade V patients: AUD 64,645.40 (IQR 42,603.10–‍99,931.90) and AUD 36,305.20 (IQR 23,680.00–55,563.20), respectively. Every cost variable significantly increased (*p* <.0001), including readmission costs (*p* =.0032). All cost variables for the severity of complications are reflected in Fig. 2 and supplementary Table 6 (see additional file 6).

There were no differences observed in the total hospital costs for patients who presented for elective and emergency surgeries: AUD 25,553 (IQR 19,375.4–38,891.5) vs. AUD 26,592.9 (IQR 18,313.6–43,378.2), respectively (*p* =.753). However, elective surgical patients who developed any complication incurred a greater hospital cost than elective patients who had no complications AUD 30,174.1 (IQR 21,710.7–47,244.7) vs. AUD 19,975.4 (IQR 15802.8–25,306.6); *p* <.0001. Similarly, emergency surgical patients who developed any complication incurred a greater hospital cost than emergency patients who had no complications AUD 29,596.7 (IQR 20,384.2–48,672.1) vs. AUD 17,809.0 (IQR 13,692.6–23,427.5); *p* <.0001.

The hospital costs of emergency and elective patients who had 0–2 complications was AUD 19,652.6 (IQR 15,342.2–26,142.9) and AUD 22,656.8 (IQR 18,008.3–31,110.7), respectively (*p* <.0001). The hospital costs of emergency and elective patients who had three or more complications was AUD 38,516.9 9 (IQR 28,126.1–62,752.1) and AUD 45,129.4 (IQR 27,684.4–67513.2), respectively (*p* =.398).

The hospital costs of emergency and elective patients who developed no complication or a minor complication (i.e., Clavien-Dindo Grades 0–2) was AUD 22,134.3 (IQR 17,065.2–30,419.0) and AUD 23,787.0 (IQR 18,503.2–33,855.6), respectively (*p* =.004). The hospital costs of emergency and elective patients who developed major complications (Clavien-Dindo Grades > 2) was AUD 50,320.3 (IQR 33,599.9–87,134.6) and AUD 59614.3 (IQR 39,337.0–88,521.7), respectively (*p* =.071). The costs of emergency and elective surgery and the association with number of complications and severity of complications is presented in Supplementary Fig. 1 (see additional file 7). Intensive care unit (ICU) and ward cost per day and anaesthetic and theatre cost per hour in patients with complications are presented in the supplementary Fig. 2 (see additional file 8). The allied health costs in patients with complications is presented in the supplementary Fig. 3 (see additional file 9).

## Discussion

This single-centre retrospective study demonstrated that 7 out of 10 patients developed postoperative complications after a major abdominal surgical procedure. Most of these complications (56.9%) were classified as minor (CVD Grades I and II). There was a significant increment in the median adjusted total hospital costs as both the number and severity of complications increased. These results are congruent with our hypotheses.

Whilst many studies have reported high rate of postoperative complications following major abdominal surgery, they often focused on a singular surgical procedure (i.e., hepatic resection), or measured one complication of interest (i.e., postoperative infections) [8–12]. This study, which comprehensively reports all postoperative complications across multiple general surgical specialties is not directly comparable with the available literature.

The development of one, two or three complications were associated with an additional median cost of AUD 2618.30 (13% increase), AUD 3605.50 (16.2% increase) and AUD 3173 (12.3% increase), respectively. Further, an exponential spike of AUD 23,719.70 (81.7% increase) in costs was evident in patients who developed more than three complications. This group accounted for 35.3% of the total hospital costs across the entire patient cohort. However, examining the data revealed that the type of surgical procedure has a vital role in the overall effects on the costs. Despite both the liver surgeries and Whipple procedure groups developing a median of three complications each, the difference in costs associated with Whipple procedures was more than double compared to liver surgery costs. The postulated reason for this difference is longer hospital stays, which mandates the utilisation of more resources and essential treatments. Wang et al. [15] concluded that patients who underwent Whipple procedures required longer hospital stays, which was deemed a necessary cost but was compounded by the exorbitant operating costs for the procedure.

Although the majority of patients developed minor postoperative complications, the overall financial impact imposed on our healthcare system by the group of patients who developed major complications amounted to 69% of the overall adjusted median total cost. Notably, as the severity of complications increased, the median adjusted hospital costs also increased, with an exponential rise of AUD 21,182.70 (71.8%) from CVD Grade II to Grade III. This is consistent with the findings of Lee et al. [13], which showed that major complications (CVD grade III and IV) were associated with an exponential increase in cost. Nevertheless, our results demonstrated that the cumulative number of minor complications also has a major cost impact for the healthcare system. It is imperative to identify patients’ factors that have a strong potential to engender postoperative complications pertaining to the complication count and severity, and ultimately, healthcare costs.

This study identified four parameters of the hospital cost bucket where each variable experienced a tremendous spike as the complication count and severity increased. These parameters were ICU, operating theatre, radiology and ward costs. This is probably due to additional resources, such as surgical intervention, longer ICU and hospital stays and imaging interventions to investigate the source of complication(s). According to Straatman et al. [14], the development of major complications necessitates resources, which markedly increases the overall costs. It is of paramount importance that major complications warrant risk stratification, quality treatment and intervention controls [13, 14].

The strengths of this study include an exhaustive, comprehensive, and descriptive analysis of the total adjusted hospital costs associated with postoperative complications. The study included all major and minor complications and analysed the relationship between the number and severity of complications and the overall healthcare cost.

## Conclusions

The high prevalence of postoperative complications following major abdominal surgeries inflicts significant financial strains on our healthcare system. These findings provide valuable data for prospective studies to investigate the relationships between a priori selected perioperative variables and the presence of complications and costs, and to assess perioperative interventions to reduce complications count and severity. In turn, this could translate into significant cost-effective and economic benefits for a heavily taxed Australian healthcare system.

## Limitations

This study has several limitations. As with many retrospective studies, selection and information bias is inevitable. However, the risk of such bias was minimised by multiple researchers crosschecking and validating the data processed in the institution’s electronic medical records. Accurate crystalloid fluid data, urine output and blood loss were not accurately reported in the medical records for many patients, especially in the postoperative period. Most of these patients did not have urinary catheters and were allowed unrestricted oral fluids as part of our institutions postoperative ERAS protocols. Further, this study was only conducted at a single institution, which can curtail validity to other centres and countries. However, the hospital ensures homogeneity in treatment protocols and upholds the highest standards consistent with other tertiary universities centres. Additionally, the major abdominal surgeries in this study consisted of four distinct surgical procedures; hence, the nature of a procedure could produce inconsistent results compared to other procedures and hinder the accuracy of the findings. Finally, multivariate analysis was not used to evaluate the factors independently associated with the development of complications, as this is part of a separate study involving a larger dataset, however we provide a detailed overview of our unique and granular cost data that can be used by other researchers for comparative health costing work.

### Electronic supplementary material

Below is the link to the electronic supplementary material.

Supplementary Material 1

Supplementary Material 2

Supplementary Material 3

Supplementary Material 4

Supplementary Material 5

Supplementary Material 6

Supplementary Material 7

Supplementary Material 8

Supplementary Material 9

Supplementary Material 10

## Data Availability

The datasets analyzed for this study are available from the corresponding upon reasonable request.

## References

[1] World Health Organization. Global spending on health: Weathering the storm [Internet]. Geneva (Switzerland); 2020 Dec 10. [cited 2023 May 19]. Available from: https://www.who.int/publications/i/item/9789240017788.

[2] Australian Institute of Health and Welfare. Health Expenditure Australia 2018-19, Canberra ACT.; 2020 Nov 06. [cited 2023 May 19]. Available from: https://www.aihw.gov.au/reports/health-welfare-expenditure/health-expenditure-australia-2018-19/contents/summary.

[3] Louis M, Johnston SA, Churilov L, Ma R, Christophi C, Weinberg L (2021). Financial burden of postoperative complications following colonic resection: a systematic review. Med (Baltim).

[4] Straatman J, Cuesta MA, Gisbertz SS, Van der Peet DL (2014). Value of a step-up diagnosis plan: CRP and CT-scan to diagnose and manage postoperative complications after major abdominal surgery. Rev Esp Enferm Dig.

[5] National Blood Authority Australia. Patient Blood Management Guidelines: Module 2, Canberra ACT.; 2011 Nov 15. [cited 2023 May 19]. Available from: https://www.blood.gov.au/system/files/documents/pbm-module-2.pdf.

[6] Agha R, Abdall-Razak A, Crossley E, Dowlut N, Iosifidis C, Mathew G, STROCSS Group (2019). STROCSS 2019 Guideline: strengthening the reporting of cohort studies in surgery. Int J Surg.

[7] Dindo D, Demartines N, Clavien PA (2004). Classification of surgical complications: a new proposal with evaluation in a cohort of 6336 patients and results of a survey. Ann Surg.

[8] Scott JW, Olufajo OA, Brat GA (2016). Use of National Burden to define operative emergency general surgery. JAMA Surg.

[9] Jakhmola CK, Kumar A (2014). Whipple’s pancreaticoduodenectomy: outcomes at a tertiary care hospital. Med J Armed Forces India.

[10] Karim SAM, Abdulla KS, Abdulkarim QH, Rahim FH (2018). The outcomes and complications of pancreaticoduodenectomy (Whipple procedure): Cross sectional study. Int J Surg.

[11] Yeung DE, Peterknecht E, Hajibandeh S, Hajibandeh S, Torrance AW (2021). C-reactive protein can predict anastomotic leak in colorectal surgery: a systematic review and meta-analysis. Int J Colorectal Dis.

[12] Jin S, Fu Q, Wuyun G, Wuyun T (2013). Management of post-hepatectomy complications. World J Gastroenterol.

[13] Lee DK, Frye A, Louis M, Koshy AN, Tosif S, Yii M (2020). Postoperative complications and hospital costs following small bowel resection surgery. PLoS ONE.

[14] Straatman J, Cuesta MA, de Lange-de Klerk ES, van der Peet DL (2015). Hospital cost-analysis of complications after major abdominal surgery. Dig Surg.

[15] Wang J, Ma R, Eleftheriou P, Churilov L, Debono D, Robbins R (2018). Health economic implications of complications associated with pancreaticoduodenectomy at a University Hospital: a retrospective cohort cost study. HPB (Oxford).

