# Supplementary material for: The hospital costs of complications following major abdominal surgery: a retrospective cohort study
Source: BMC Res Notes. 2024 Feb 27;17:59. doi: 10.1186/s13104-024-06720-z (PMC10900687; doi:10.1186/s13104-024-06720-z)
Supplement: Supplementary file 1 — Supplementary Material 1 [file 13104_2024_6720_MOESM1_ESM.pdf]

Supplementary Table 1. Patient baseline characteristics and preoperative data. Data is presented as median (Interquartile range), Mean (SD), Min – Max values, number (proportion).

Variables		Total (n= 1790)	Colorectal (n= 868)	Liver (n=422)	Small bowel (n=348)	Whipple's (n=152)
**Sex:**	**Male**	1012 (56.5%)	458 (52.8%)	251 (59.5%)	198 (56.9%)	105 (69.1%)
	**Female**	778 (43.5%)	410 (47.2%)	171 (40.5%)	150 (43.1%)	47 (30.9%)
**Age (years)**		64 (53:74); 18-98	66 (55:76); 18-96	60 (50-67); 18-85	65 (51:78); 18-98	66 (56:72); 18-85
**Height (cm)**		167 (161:175); 140-206	167 (160:175); 140-19	169 (162:175); 142-206	168 (160:175); 147-195	167 (160:175); 144-188
**Weight (kg)**		74 (63:86.5); 35-156	75 (63:86); 35-155	76 (64:90); 42-146	71 (60:84); 36-156	75 (64:82); 43-144
**Body Mass Index (kg/m^2)**		26 (23:30); 14-56	26 (23:30); 15-50	26 (23:30); 16-54	25 (22:29); 14-55	26 (23:28.3); 16-56
**American Society Anaesthesiology Class:**	**I**	78 (4.4%)	45 (5.2%)	11 (2.6%)	21 (6.0%)	1 (0.7%)
	**II**	669 (37.4%)	363 (41.8%)	142 (33.7%)	115 (33.1%)	49 (32.2%)
	**III**	876 (48.9%)	383 (44.1%)	257 (60.9%)	140 (40.2%)	96 (63.2%)
	**IV**	160 (8.9%)	73 (8.4)	12 (2.8%)	69 (19.8%)	6 (3.9%)
	**V**	7 (0.4%)	4 (0.5%)	0	3 (0.9%)	0
**Combined Complication Index**		20 (8:32); 0-139	17 (0:30); 0-100	12 (0:24); 0-193	24 (8:39); 0-126	22 (12:34); 0-100
**Malignancy**		1098 (61.3%)	567 (65.3%)	353 (83.7%)	58 (16.7%)	120 (78.9%)
**Emergency surgery**		529 (29.5%)	255 (29.3%)	9 (2.1%)	251 (72.1%)	14 (9.2%)
**Preop chemotherapy within 3 months**		186 (10.4%)	75 (8.6%)	73 (17.3%)	15 (4.3%)	23 (15.1%)

**Previous resection**	195 (11.2%)	65 (7.9%)	38 (9.0%)	92 (26.4%)	23 (15.1%)
**Smoker within 1 year**	367 (20.5%)	187 (21.5%)	88 (20.8%)	14 (4.0%)	0
**Alcohol** misuse	105 (5.9%)	187 (21.5%)	88 (20.8%)	60 (17.2%)	32 (21.0%)
**Charlson Comorbidity Index** (CCI)	5 (3:8); 0-15	6 (4:8); 0-14	6 (4:8); 0-14	4 (2:6); 0-15	5 (4:6); 2-10
**Haemoglobin (g/dl)**	133 (118:145); 65 - 189	130 (115:144); 65-189	138 (128:150); 76-182	131 (113: 144); 69-180	131 (118:141); 79-173
**Anaemia (WHO definition)**	626 (35.0%)	363 (41.8%)	71 (16.8%)	140 (40.2%)	52 (34.2%)
**Ferritin (units)**	154 (70:255); 4-5313	131 (60:217); 5-3450	177 (93:278); 5-1581	139 (62:282); 8-5313	261 (145:418); 4-2603
**Transferrin saturation (%)**	20 (14:24); 2-189	18 (13:23); 2-121	22 (18:28); 3-118	18 (11.8 – 26.3); 2-189	21 (17:23); 4-44
**White cell count (x 10^9/L)**	7 (5:9); 1-147	7 (5:9); 1-42	6 (5:8); 1-93	9 (6:13); 2-42	7 (5:9); 2-147
**Platelet count (x10^9/L)**	253 (200:313); 18-887	258.8 (209.3:323.8); 24-801	236.5 (182.3:269); 18-628	251.5 (199.8:330.3); 59-887	281 (214: 341); 112-871
**Creatinine (mg/dL)**	75 (63:90); 7-931	75 (63:92); 25-931	75 (64:84); 38-298	77 (62:100); 36-398	69 (58:83); 7-241
**Estimated Glomerular Filtration Rate (mL/min/1.73m^2)**	82 (68:91); 4-101	83 (67:91); 4-92	83 (76:90); 16-101	68 (47.5:81.8); 10-90	86 (74:90); 23-91
**Albumin (g/dL)**	36 (32:39); 8-83	36 (32:39); 8-49	37 (36:39); 19-46	34 (28:38); 14-48	36 (31.3: 39.8); 19-83
**Bilirubin (mg/dL)**	9 (5:14); 1-411	7 (5:10); 1-80	11 (8:16); 1-325	9 (5:14); 2-130	14.5 (7:41); 3-411
**International Normalised Ratio**	1.1 (1:1.1); 0.8-5.4	1.1 (1:1.1); 0.8-3.4	1.1 (1:1.1); 0.9-5.4	1.1 (1:1.3); 0.9-4.7	1.1 (1:1.1); 0.9-1.6
**Urea (mg/dL)**	5 (4:7); 1- 16	0	0	0	5 (4:7); 1-16
