# Supplementary material for: The hospital costs of complications following major abdominal surgery: a retrospective cohort study
Source: BMC Res Notes. 2024 Feb 27;17:59. doi: 10.1186/s13104-024-06720-z (PMC10900687; doi:10.1186/s13104-024-06720-z)
Supplement: Supplementary file 2 — Supplementary Material 2 [file 13104_2024_6720_MOESM2_ESM.pdf]

Supplementary Table 2. Intraoperative variables. Data is presented as median (interquartile range), Minimum – Maximum values, number (proportion).

Variables	Total (n=1790)	Colorectal (n=868)	Liver (n =422)	Small bowel (n=348)	Whipple's (n=152)
**Total intravenous anaesthesia**	718 (40.1%)	390 (45%)	106 (25.1%)	160 (45.9%)	62 (40.8%)
**Volatile anaesthesia**	556 (31%)	312 (36%)	71 (16.8%)	138 (39.6%)	35 (23%)
**Combination TIVA and volatile**	448 (25.0%)	166 (19.1%)	177 (41.9%)	50 (14.4%)	55 (36.2%)
**Epidural anaesthesia**	9 (0.5%)	0	1 (0.2%)	8 (2.3%)	0
**Spinal anaesthesia**	112 (6.3%)	0	88 (20.9%)	24 (6.9%)	0
**Rectus sheath block**	15 (0.8%)	0	2 (0.5%)	13 (3.7%)	0
**Transversus abdominis plane block**	41 (2.3%)	0	2 (0.5%)	39 (11.2%)	0
**Intercostal nerve block**	2 (0.1%)	0	0 (0)	2 (0.6%)	0
**Duration of surgery (minutes)**	260 (195:345); 16 - 1028	270 (212:325); 16 - 810	249 (181.8:355); 60 - 920	197 (160.3:249.8); 45 - 655	489 (416.3:605.3); 225 - 1028
**Urine output (ml/day)**	560 (365:920); 0 - 3510	Not recorded	Not recorded	Not recorded	560 (365:920); 0 - 3510
**Blood loss (mL)**	350 (250:600); 0 - 40000	Not recorded	550 (400:1825); 100 - 40000	Not recorded	300 (250:400); 0 - 3500
**Patients receiving blood (RBC)**	84 (4.7%)	22 (2.5%)	37 (8.8%)	24 (6.9%)	1 (0.7%)

**Median number of red blood cells administered**		2 (1:3); 1 - 20	1 (1:2); 1 - 4	2 (2:4); 1 - 18	1.5 (1 - 2.3); 1 - 20	1 (1:1); 1 - 1
**Fresh frozen plasma (units)**		16 (0.9%); 0 - 10	Not recorded	5 (1.2%); 0 - 4	11 (3.2%); 0 - 10	0
**Platelets (units)**		7 (0.4%); 0 - 4	Not recorded	3 (0.7%); 0 - 4	4 (1.1%); 0 - 2	0
**Cryoprecipitate (units)**		3 (0.2%); 0 - 30	Not recorded	2 (0.5%); 0 - 30	1 (0.3%); 0 - 8	0
**Crystalloids (mL)**		2000 (1000:2375); 0 - 10000	Not recorded	2000 (1000:3000); 0 - 10000	2000 (1000:2000); 0 - 6000	3250 (2500:4000) 1000 - 12000
**4% Albumin (mL)**		0 (0:0); 0 - 2000	Not recorded	0 (0:0); 0 - 2000	0 (0:0); 0 - 200	0 (0:0); 0 - 200
**20% Albumin (mL)**		0 (0:0); 0 - 3200	Not recorded	0 (0:200); 0 - 3200	0 (0:0); 0 - 600	200 (0: 500); 200 - 2000
**Concomitant procedure**		296 (17.6%)	117 (13.5%)	141 (44.6%)	17 (4.9%)	21 (13.8%)
**Procedure type**	Laparoscopic	637 (37.8%)	513 (59.1%)	89 (28.2%)	35 (10.1%)	0
	Laparoscopic assisted	55 (3.3%)	30 (3.5%)	0	25 (7.2%)	0
	Laparoscopic converted to Open	141 (8.4%)	80 (9.2%)	19 (6.0%)	42 (12.1%)	0
	Open	851 (50.5%)	245 (28.2%)	208 (65.8%)	246 (70.7%)	152 (100%)
