# Supplementary material for: The hospital costs of complications following major abdominal surgery: a retrospective cohort study
Source: BMC Res Notes. 2024 Feb 27;17:59. doi: 10.1186/s13104-024-06720-z (PMC10900687; doi:10.1186/s13104-024-06720-z)
Supplement: Supplementary file 3 — Supplementary Material 3 [file 13104_2024_6720_MOESM3_ESM.pdf]

Supplementary Table 3. Postoperative variables. Data is presented as median (25th:75th), Mean (SD), Min – Max values, number (proportion).

Variables	Total (n=1790)	Colorectal (n=868)	Liver (n=422)	Small bowel (n=348)	Whipple's (n=152)
**Day 1 Hb (mg/dL)**	113 (99:126); 53-172	115 (101:128); 53-172	113 (97.5:124); 67-163	113.5 (99:126.3); 68-165	105 (92:115.8); 67-139
**Lowest Hb within 7 days (mg/dL)**	101 (86:113); 4-163	103 (91:116); 53-162	98.5 (84:113.3); 54-154	99 (83.5:113.5); 57-163	88.5 (79:99); 4-128
**Day 1 Fluid Balance (mL)**	1085 (569.3:1939); 14-3304	Not recorded	Not recorded	Not recorded	1085 (569.3:1939); 14-3304
**Day 1 Crystalloids (mL)**	2297 (1574:2800); 198-5996	Not recorded	Not recorded	Not recorded	2297 (1574:2800); 198-5996
**Day 1 Colloids (mL)**	0 (0:287.5); 0-1500	Not recorded	Not recorded	Not recorded	0 (0:287.5); 0-1500
**Number of patients receiving blood**	308 (17.2%)	148 (17.1%)	57 (13.5%)	72 (20.7%)	31 (20.4%)
**Median number of red blood cells administered**	2 (1:4); 1 – 39	2 (1:3); 1 - 12	2 (2:5); 1 - 30	2 (1:5); 1 - 39	2 (1:5); 1 - 13
**Number of patients admitted to ICU**	840 (46.9%)	266 (30.6%)	291 (69%)	132 (37.9%)	151 (99.3%)
**Intensive Care Unit Time (hours)**	37.5 (14:91.3); 3-1048	52.5 (19.3:134.3); 3-877	16 (12:42.5); 4-490	100 (35.8: 174); 5-1048	42.5 (17.3:68); 1-297
**Hospital length of stay (days)**	8 (6:13.25); 1-199	8 (6:13); 1-199	6 (5:8); 1-142	9 (6:17); 1-189	12 (9:21.8); 2-7
**Readmission within 30 days**	251 (14.0%)	131 (15.1%)	42 (9.9%)	50 (14.4%)	28 (18.4%)
**In hospital death**	50 (2.8%)	22 (2.5%)	2 (0.5%)	23 (6.6%)	3 (2.0%)
