# Supplementary material for: The hospital costs of complications following major abdominal surgery: a retrospective cohort study
Source: BMC Res Notes. 2024 Feb 27;17:59. doi: 10.1186/s13104-024-06720-z (PMC10900687; doi:10.1186/s13104-024-06720-z)
Supplement: Supplementary file 4 — Supplementary Material 4 [file 13104_2024_6720_MOESM4_ESM.pdf]

Supplementary Table 4. Number and severity (Clavien-Dindo Grade) of complications of patients undergoing abdominal surgery. Data is presented as median (25th:75th), Mean (SD), Min – Max values, number (proportion).

Variables		Total (n=1790)	Colorectal (n=868)	Liver (n=422)	Small Bowel (n=348)	Whipple's (n=152)
**Complication**		1346 (75.2%)	632 (72.8%)	304 (72.0%)	283 (81.3%)	127 (83.5%)
**No. of complications**		2 (0:3)	2 (0:3)	1 (0:3)	3 (1:4)	3 (1:4)
**No. of complications grouped**	0	444 (24.8%)	236 (27.2%)	118 (28.0%)	65 (18.7%)	25 (16.4%)
	1	386 (21.6%)	197 (22.7%)	109 (25.8%)	59 (17%)	21 (13.8%)
	2	313 (17.5%)	169 (19.5%)	68 (16.1%)	48 (13.8%)	28 (18.4%)
	3	230 (12.8%)	99 (11.4%)	49 (11.6%)	56 (16.1%)	26 (17.1%)
	4+	417 (23.3%)	167 (19.2%)	78 (18.5%)	120 (34.5%)	52 (34.2%)
**Clavien-Dindo Classification (Grade)**	0	444 (24.8%)	236 (27.2%)	118 (28.0%)	65 (18.7%)	25 (16.4%)
	I	410 (22.9%)	200 (23.0%)	123 (29.1%)	59 (17.0%)	28 (18.4%)
	II	608 (34.0%)	268 (30.9%)	129 (30.6%)	137 (39.4%)	74 (48.7%)
	III	108 (6.0%)	47 (5.4%)	24 (5.7%)	21 (6.0%)	16 (10.5%)
	IV	170 (9.5%)	95 (10.9%)	26 (6.2%)	43 (12.4%)	6 (3.9%)
	V	50 (2.8%)	22 (2.5%)	2 (0.5%)	23 (6.6%)	3 (2.0%)
