# Supplementary material for: The hospital costs of complications following major abdominal surgery: a retrospective cohort study
Source: BMC Res Notes. 2024 Feb 27;17:59. doi: 10.1186/s13104-024-06720-z (PMC10900687; doi:10.1186/s13104-024-06720-z)
Supplement: Supplementary file 5 — Supplementary Material 5 [file 13104_2024_6720_MOESM5_ESM.pdf]

Supplementary Table 5. Relationship between number of complications and costs. Cost in Australian dollar ($).

Cost variables	0 Complications	1 Complication	2 Complications	3 Complications	>3 Complications	P Value
**Total Costs ($)**	19632.4 (15174.1:25017.3); 4477.4 – 116740.4	22250.7 (17668.3:29595.9); 7123.4 – 170158.4	25856.2 (20632.3:35250.1); 9780.6 – 157926120	29030 (22407.3:43489.9); 10681.4 – 150879.5	52749.7 (33112.6:80086.1); 13688 – 629137.2	p<0.0001
**Allied health cost ($)**	339.7 (13.2:511.2); 0 – 4589.6	464.5 (31.2: 958.2); 0 – 4688.4	511.7 (196.9:1172.3); 0 – 4256.1	891.2 (435.5:1494.4); 7.5 – 3441.7	1349.3 (736.9:2083); 0 – 17963.2	p<0.0001
**Anaesthesia cost ($)**	2427.2 (1737.4:3284.1); 0 – 20529.5	2667.5 (1820:3424.6); 0 – 24412.3	2697.9 (1826.1:3573.5); 0 – 20951.3	2824.2 (1983.7:3833.4); 0 – 18298	3323.7 (2174.7:4791); 0 – 27756.9	p<0.0001
**Blood product cost ($)**	0 (0:0); 0 – 3303	0 (0:0); 0 – 18207.8	0 (0:24.5); 0 – 8368.9	0 (0:130.9); 0 – 14234	137.8 (0:1296.5); 0 – 37011.4	p<0.0001
**Health in the home cost ($)**	0 (0:0); 0 – 10748.4	0 (0:0); 0 – 7075.2	0 (0:0); 0 – 9451.1	0 (0:0); 0 – 12285.1	0 (0:0); 0 – 37662.9	p<0.0001
**Intensive care unit cost ($)**	0 (0:545.3); 0 – 14437	0 (0:1803.4); 0 – 41794.8	0 (0:2111.2); 0 – 43828.9	0 (0:3042.5); 0 – 37562	5829.6 (0:17844.3); 0 – 183974.1	p<0.0001
**Medical cost ($)**	1279 (938.8:1686.9); 130.6 – 13381.7	1625.1 (1158.2:2376.0); 155.5 – 10012.7	1970.1 (1456.7:2858.7); 697.3 – 13735.0	2342.3 (1675.7:3294); 507.2 – 24880.5	4038.3 (2285.2:6827); 218.5 – 78920.4	p<0.0001
**MET Call cost ($)**	0 (0:0); 0 – 586.1	0 (0:0); 0 – 916.5	0 (0:0); 0 - 841.8	0 (0:0); 0 – 976.9	0 (0:196.1); 0 – 2131.5	p<0.0001
**Operating theatre cost ($)**	8459.3 (6167.5:11585.6); 0 – 51086.9	8690.9 (6206.2:12227.3); 1460.2 – 47729.3	9365.5 (6773.7:13152); 0 – 115504.7	8512.4 (5968:12421.2); 0 – 62055.8	10281.4 (6701.4:16147.8); 0 – 92332.6	p<0.0001
**Pathology cost ($)**	733.4 (405:1162.4); 0 – 6228.7	798.7 (453.6:1206.4); 0 – 6918.9	1008.3 (566.3:1361.6); 0 – 5588.7	1017.1 (624.3:1505.6); 0 – 4510.8	1740.1 (1095:2666); 0 – 15566.2	p<0.0001
**Pharmacy cost ($)**	295.8 (178:405.6); 0 – 6285.5	313.2 (197.3:446.6); 0 – 3537.5	349.8 (248.5:575.4); 0 – 3301.2	476 (329:765); 0 – 24233.6	853.4 (462.6:2513.2); 0 – 79377.9	p<0.0001

**Radiology cost ($)**	0 (0:153); 0 – 2035.8	111.89 (0:446.8); 0 – 5593.1	196.9 (70.1:763.9); 0 – 5061.1	340.2 (98.3:1052.2); 0 – 4453.9	1514.9 (669.2:2648.6); 0 – 25333.3	$p<0.0001$
**Readmission cost ($)**	0 (0:0); 0 – 67610.5	0 (0:0); 0 – 38948	0 (0:0); 0 – 110217.7	0 (0:0); 0 – 50136.3	0 (0:0); 0 – 195896.9	$p=0.0068$
**Ward cost ($)**	4374 (3471.1:5520.5); 100.9 – 24676.3	5143.2 (4079.9:6654.4); 70.1 – 34612.1	6289 .4 (5046.6:9015.7); 130 – 40881.1	7956.5 (5667:10324.7); 163.7 – 36117.3	11453.2 (7351.4:17973.8); 66.3 – 222883.6	$p<0.0001$
