# Supplementary material for: The hospital costs of complications following major abdominal surgery: a retrospective cohort study
Source: BMC Res Notes. 2024 Feb 27;17:59. doi: 10.1186/s13104-024-06720-z (PMC10900687; doi:10.1186/s13104-024-06720-z)
Supplement: Supplementary file 6 — Supplementary Material 6 [file 13104_2024_6720_MOESM6_ESM.pdf]

Supplementary Table 6. Relationship between severity (Clavien-Dindo Grade) of complications and costs. Cost in Australian dollar ($).

Cost variables	Clavien-Dindo Grade I	Clavien-Dindo Grade II	Clavien-Dindo Grade III	Clavien-Dindo Grade IV	Clavien-Dindo Grade V	P value
**Total Costs ($)**	21903.6 (17663.6:27742); 7123.4 – 150879.5	29519.7 (21828.8:40527.9); 8320.2 – 320191.3	50702.4 (35866:69296.8); 19607.4 – 477157	64645.4 (42603.1:99931.9); 13443.3 – 629137.2	36305.2 (23680:55563.2); 9647.8 – 321404.5	p<0.0001
**Allied health cost ($)**	462.1 (24.3:955.2); 0 – 2853.9	888.2 (406.4:1498.4); 0 – 11938.7	1022.7 (554.8:1920.9); 32.5 – 6969.8	1561.1 (907.3:2263.1); 10.7 – 17963.2	818.9 (193.1:1506.2); 0 – 3455.8	p<0.0001
**Anaesthesia cost ($)**	2597.1 (1745.6:3459.2); 0 – 20951.3	2864.7 (1933.4:3706.9); 0 – 24412.3	3834.3 (2556:5123.9); 0 – 27756.9	3257.4 (2201.1:5161.9); 0 – 17671.9	2695.5 (1940.9:3859.5); 0 – 14256.1	p<0.0001
**Blood product cost ($)**	0 (0:0); 0 – 9034.4	0 (0:150.3); 0 – 35671.9	0 (0:405); 0 – 36404.2	530.2 (0:2146.1); 0 – 37011.4	149.2 (0:1598.5); 0 – 14721.4	p<0.0001
**Health in the home cost ($)**	0 (0:0); 0 – 5864.9	0 (0:0); 0 – 15686.1	0 (0:793.6); 0 – 37662.9	0 (0:0); 0 – 20648	0 (0:0); 0 – 2123.8	p<0.0001
**Intensive care unit cost ($)**	0 (0:1307.2); 0 – 29104.4	0 (0:2508.8); 0 – 171157.4	1748.8 (0:5774); 0 – 110681.4	16602.7 (9135.3:31660.2); 0 – 183974.1	10180.2 (2740.5:21136.2); 0 – 183088.9	p<0.0001
**Medical cost ($)**	1594.5 (1137.2:2211.9); 396.3 – 22770.9	2363.4 (1623.3:3410.1); 218.5 – 27464.8	4823 (3006:7354.4); 1305.7 – 78920.4	5034.9 (2520.4:7917); 711.7 – 50354.7	1346.1 (663.5:3394.2); 155.5 – 25325.4	p<0.0001
**MET Call cost ($)**	0 (0:0); 0 – 232.8	0 (0:0); 0 – 1379.9	0 (0:0); 0 – 1147.8	0 (0:208.8); 0 – 2131.5	0 (0:213.2); 0 – 2130.1	p<0.0001
**Operating theatre cost ($)**	8998.7 (6338.3:12400.9); 0 – 62055.8	8816.6 (6411.5:13251.1); 0 – 115504.7	13206.7 (9278:16247.4); 3385 – 69621	9654.2 (6149.6:15229.8); 0 – 92332.6	8843.9 (5077.1:13186.9); 2935.5 – 59121.7	p<0.0001
**Pathology cost ($)**	787.3 (451.9:1243.8); 0 – 5588.7	1069.2 (642.4:1531.6); 0 – 9656.9	1824.7 (1209:2607.2); 0 – 13922.4	1844.3 (1120.7:3195); 0 – 15566.2	1332.2 (674.5:2314.3); 21.9 – 7768.3	p<0.0001
**Pharmacy cost ($)**	317.4 (204.8:421.4); 0 – 2644.6	454.1 (287.4:809.6); 0 – 18779.7	749 (515.1:2418.2); 94.3 – 22640.6	935.1 (512.6:3339.1); 71.5 – 79337.9	410 (9.1: 1497.4); 0 – 17693.6	p<0.0001

**Radiology cost ($)**	95.7 (0:350.8); 0 – 3722	351.7 (98.1:1042); 0 – 8536.3	1863.1 (1030.7:3156.5); 0 – 14131.3	1683 (506.2:3580.4); 0 – 25333.3	604.6 (111.3:2265.3); 0 – 11697.5	p<0.0001
**Readmission cost ($)**	0 (0:0); 0 – 35205	0 (0:0); 0 – 195896.9	0 (0:0); 0 – 22590.2	0 (0:0); 0 – 66319.5	0 (0:0); 0 – 0	p=0.0032
**Ward cost ($)**	5261.1 (4196.7:6825.6); 129.4 – 36117.3	7449.4 (5608.3:10637.1); 213.6 – 56503	14687.4 (9589:20287.5); 2806.4 – 78790.5	12108 (6893.6:18924.2); 2119.6 – 222883.6	1618.9 (413.8:10249.8); 65.3 – 49825.9	p<0.0001
