# Supplementary material for: The hospital costs of complications following major abdominal surgery: a retrospective cohort study
Source: BMC Res Notes. 2024 Feb 27;17:59. doi: 10.1186/s13104-024-06720-z (PMC10900687; doi:10.1186/s13104-024-06720-z)

Additional File 7. Supplementary Figure 1. Costs of emergency and elective surgery and the association with number of complications (A) and severity of complications (B). Cost in Australian Dollar (AUD$).

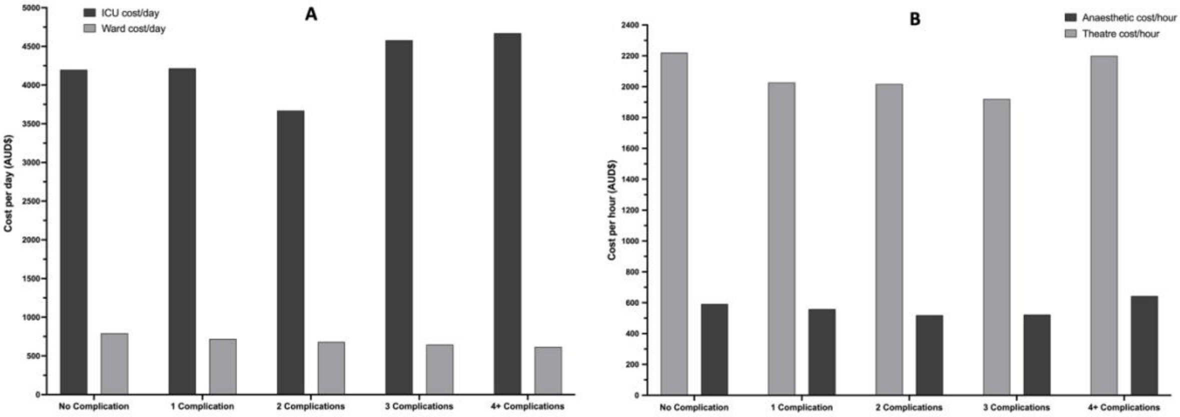

Supplement: Supplementary file 7 — Supplementary Material 7 [file 13104_2024_6720_MOESM7_ESM.pdf]
