# Supplementary material for: The hospital costs of complications following major abdominal surgery: a retrospective cohort study
Source: BMC Res Notes. 2024 Feb 27;17:59. doi: 10.1186/s13104-024-06720-z (PMC10900687; doi:10.1186/s13104-024-06720-z)

Additional File 8. Supplementary Figure 2. Intensive care unit (ICU) and ward cost per day (A), and anaesthetic and theatre cost per hour (B) in patients with complications. Cost in Australian Dollar (AUD$).

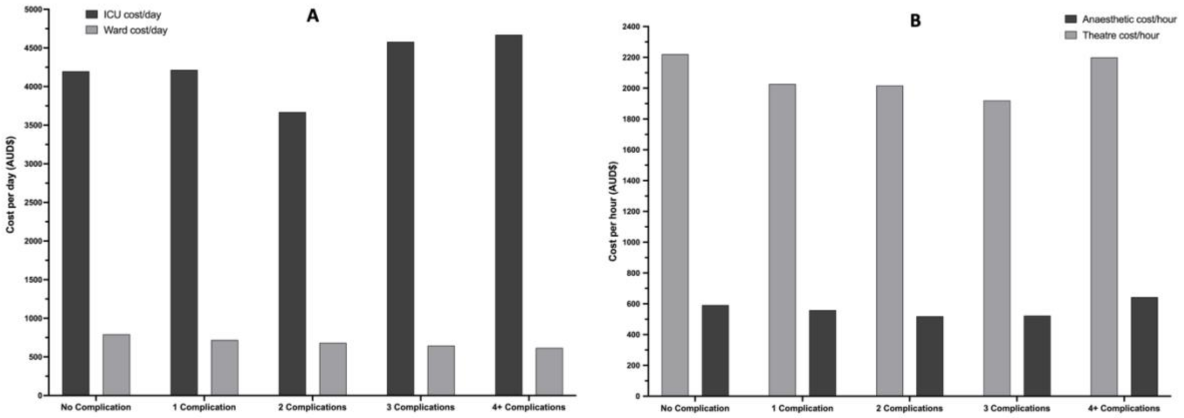

Supplement: Supplementary file 8 — Supplementary Material 8 [file 13104_2024_6720_MOESM8_ESM.pdf]
