# Supplementary material for: The hospital costs of complications following major abdominal surgery: a retrospective cohort study
Source: BMC Res Notes. 2024 Feb 27;17:59. doi: 10.1186/s13104-024-06720-z (PMC10900687; doi:10.1186/s13104-024-06720-z)

Additional File 9. Supplementary Figure 3. Allied health costs in patients with complications. Cost in Australian Dollar (AUD$).

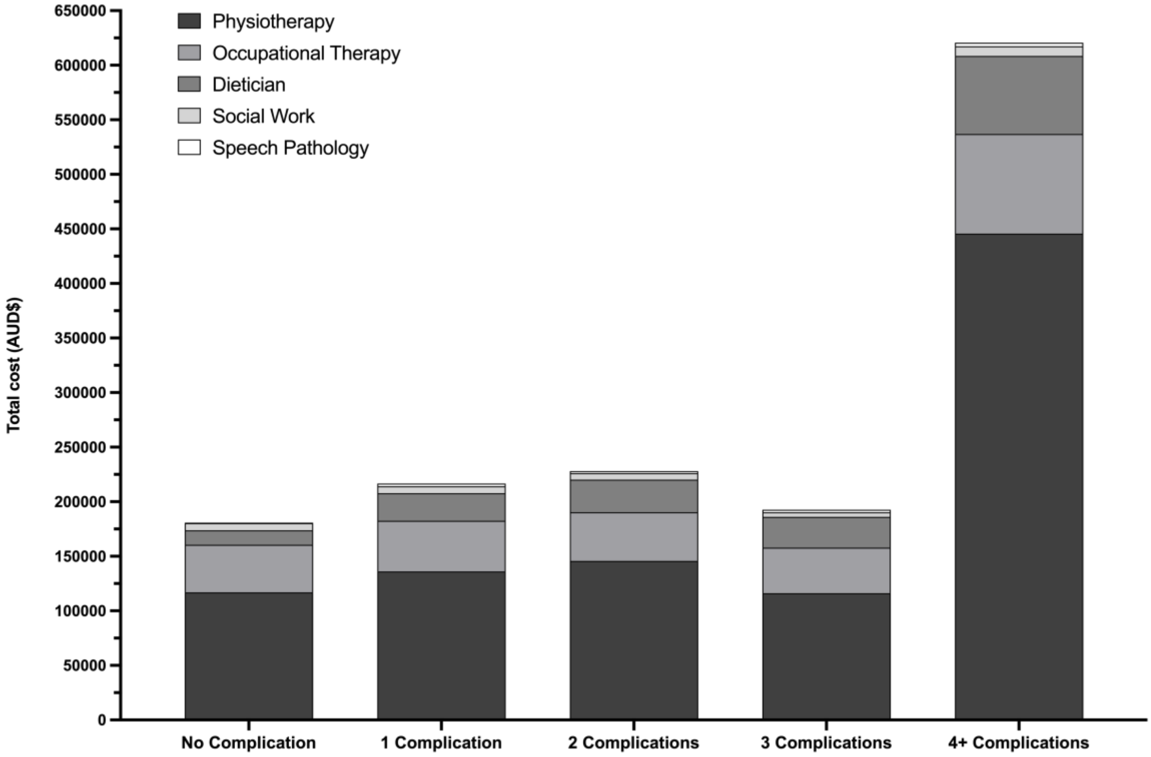

Supplement: Supplementary file 9 — Supplementary Material 9 [file 13104_2024_6720_MOESM9_ESM.pdf]
