# Supplementary material for: The hospital costs of complications following major abdominal surgery: a retrospective cohort study
Source: BMC Res Notes. 2024 Feb 27;17:59. doi: 10.1186/s13104-024-06720-z (PMC10900687; doi:10.1186/s13104-024-06720-z)
Supplement: Supplementary file 10 — Supplementary Material 10 [file 13104_2024_6720_MOESM10_ESM.docx]

**Supplementary legends**

**Additional File 1.** Supplementary Table 1**.** Patient baseline characteristics and preoperative data.

**Additional File 2.** Supplementary Table 2. Intraoperative variables.

**Additional File 3.** Supplementary Table 3. Postoperative variables.

**Additional File 4.** Supplementary Table 4. Number and severity (Clavien-Dindo Grade) of complications of patients undergoing abdominal surgery.

**Additional File 5.** Supplementary Table 5. Relationship between number of complications and costs.

**Additional File 6.** Supplementary Table 6. Relationship between severity (Clavien-Dindo Grade) of complications and costs.

**Additional File 7.** Supplementary Figure 1. Costs of emergency and elective surgery and the association with number of complications (A) and severity of complications (B). Cost in Australian Dollar (AUD$).

**Additional File 7.** Supplementary Figure 2. Intensive care unit (ICU) and ward cost per day (A), and anaesthetic and theatre cost per hour (B) in patients with complications. Cost in Australian Dollar (AUD$).

**Additional File 9.** Supplementary Figure 3. Allied health costs in patients with complications. Cost in Australian Dollar (AUD$).
